# Supplementary material for: The Integrated Stress Response Is Tumorigenic and Constitutes a Therapeutic Liability in Somatotroph Adenomas
Source: Int J Mol Sci. 2022 Oct 28;23(21):13067. doi: 10.3390/ijms232113067 (PMC9653568; doi:10.3390/ijms232113067)
Supplement: Supplementary file 1 [file ijms-23-13067-s001.zip › ijms-1918328-supplementary.pdf]

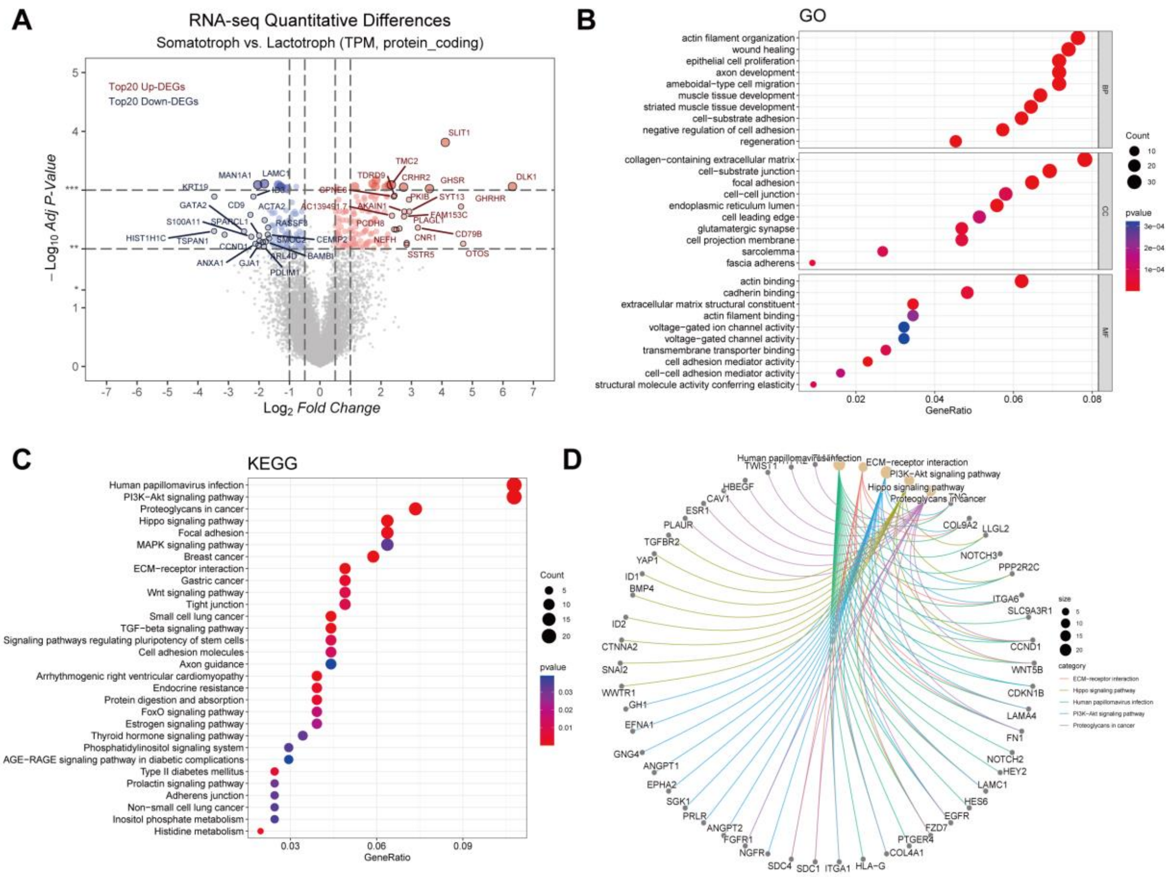

Figure S1. Transcriptome analysis of somatotroph and lactotroph adenomas. A: Volcano map of differentially expressed proteins. B: The most enriched GO terms. CD: KEGG pathway analysis based on DEGs.

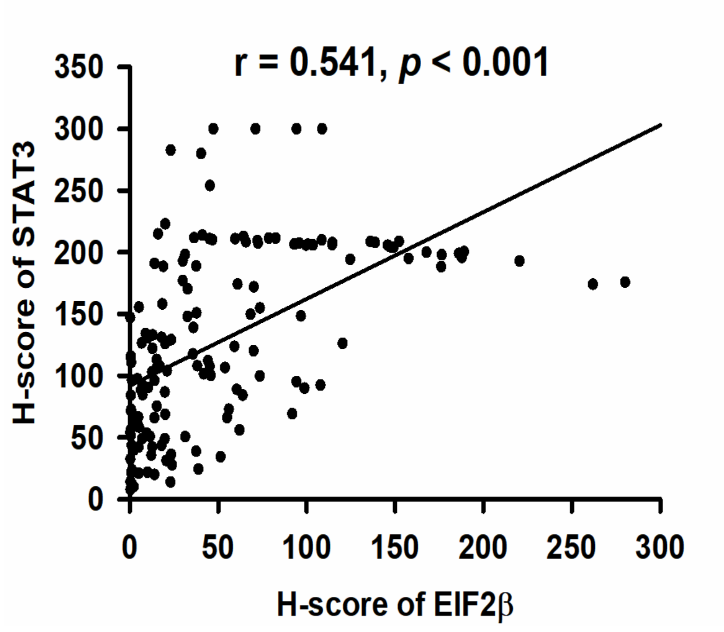

Figure S2. Correlation between EIF2β H-score and STAT3 H-score in patients with pituitary adenomas.
